# COVID-19-Omics Report: From Individual Omics Approaches to Precision Medicine

**DOI:** 10.3390/reports6040045

**Published:** 2023-09-22

**Authors:** Irina Vlasova-St. Louis, Daniel Fang, Yara Amer, Hesham Mohei

**Affiliations:** 1 Individualized Genomics and Health Program, Johns Hopkins University, Baltimore, MD 21218, USA; 2 APHL-CDC Laboratory Program, Silver Spring, MD 20910, USA; djfang99@gmail.com; 3 Department of Medicine, Al Ahrar Teaching Hospital, Zagazig 44511, Egypt; 4 Department of Pathology and Laboratory Medicine, Perelman School of Medicine, University of Pennsylvania, Philadelphia, PA 19104, USA; hesham.mohei@pennmedicine.upenn.edu

**Keywords:** COVID-19 pandemic, immune responses to SARS-CoV-2, acute respiratory distress syndrome (ARDS), genomics, transcriptomics, proteomics, epigenomics, metabolomics, microbiomics, precision and preventive medicine, public health

## Abstract

During the COVID-19 pandemic, it became apparent that precision medicine relies heavily on biological multi-omics discoveries. High throughput omics technologies, such as host genomics, transcriptomics, proteomics, epigenomics, metabolomics/lipidomics, and microbiomics, have become an integral part of precision diagnostics. The large number of data generated by omics technologies allows for the identification of vulnerable demographic populations that are susceptible to poor disease outcomes. Additionally, these data help to pinpoint the omics-based biomarkers that are currently driving advancements in precision and preventive medicine, such as early diagnosis and disease prognosis, individualized treatments, and vaccination. This report summarizes COVID-19-omic studies, highlights the results of completed and ongoing omics investigations in individuals who have experienced severe disease outcomes, and examines the impact that repurposed/novel antiviral drugs, targeted immunotherapeutics, and vaccines have had on individual and public health.

## 1. Introduction

**Figure 1:**
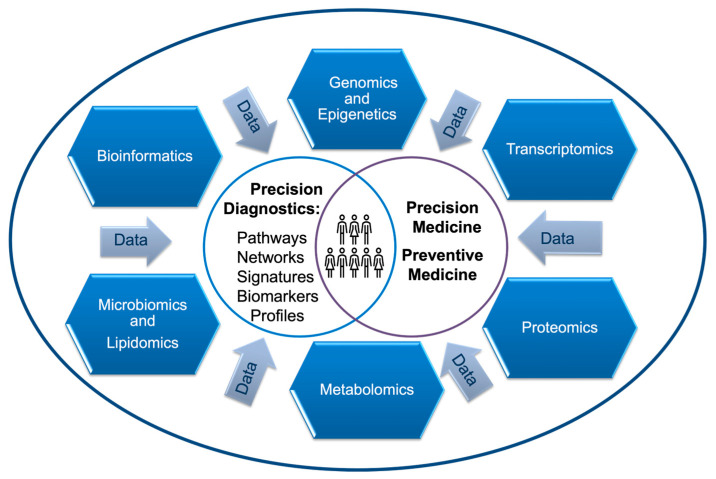
Contribution of individual omics disciplines (outlined in hexagons) to population-centric precision medicine and preventive medicine innovation. Data collected from various individual omics technologies provide comprehensive information (depicted by arrows) to allow for the identification of novel biomarkers, signatures, pathways, profiles, and networks (precision diagnostics). Omics technologies have become a part of precision diagnostic medicine, customizing population-centric treatments (precision medicine) and leading to novel vaccine development (preventive medicine).

It became apparent during the coronavirus disease of 2019 (COVID-19) pandemic that precision medicine relies heavily on high throughput technologies in multiomic biological discoveries. Rapid next-generation sequencing of SARS-CoV-2 lineages became a precision diagnostic procedure that was particularly indispensable to public health epidemiologists in understanding infection transmissibility, phylogenetic diversification, and traceable sources of infection [1,2]. High throughput single- and multiomics approaches, implemented by many university medical centers and the CDC, have aided in the discovery of viral virulence factors, mechanisms of replication, and immuno-pathogenicity [3,4]. The efficient use of systematically collected multiomic information, data analytics, and predictive modeling has allowed researchers to gain an understanding of the disease and build strategies to drive the informed diagnostic process (Figure 1). In addition to diagnosis, the prediction of the severity and prognosis of the mortality rate of COVID-19 were essential to stratifying patients according to their precise medical care needs [5]. 

Since the start of the COVID-19 pandemic, approximately 1.2 million COVID-19-associated deaths have occurred in the United States [6]. The clinical symptoms of COVID-19 vary broadly, ranging from mild cold-like symptoms to acute respiratory distress syndrome (ARDS). The epidemiology of severe clinical manifestations revealed a significant skew toward people with pre-existing medical conditions, particularly individuals with primary immunodeficiencies. Moreover, a new phenomenon, long COVID-19, has become a global public health problem [7]. Long COVID cases present with diverse multi-organ symptomatology, and long COVID-10 is nearly three times more prevalent in adults ages 50–59 [8]. 

However, demographic studies have revealed that age or co-morbidities are not the only risk factors for life-threatening outcomes or long COVID-19 [9]. Precision diagnostics, powered by omics studies, have discovered underrepresented vulnerable populations, which are summarized in this report. Comprehensive omics investigations have shed light on many previously unknown determinants of immune response to existing and novel SARS-CoV infections, representing a global scientific effort to combat this new disease. Breakthrough data, delivered via high-throughput omics technologies, have allowed biopharma to initiate the development of the precision therapeutics and vaccines that are necessary to improve the medical care-delivery process [10]. In this report, we discuss cross-omics differences underlying the immune response to SARS-CoV-2 and COVID-19 severity in various demographic populations. We summarize the contributions of COVID-19-omic studies to advancements in precision and preventive medicine (Figure 1). 

## 2. Population-Centric Approach: Demographics of COVID-19 Vulnerable Population

In the population, COVID-19 has remarkably diverse clinical pathologies, ranging from asymptomatic SARS-CoV-2 carriers to fulminant life-threatening disease and long COVID-19 [11,12]. In some instances, multiple clusters of SARS-CoV-2 positive cases without any symptoms at the time that they were tested were reported prior to vaccine implementation [13,14]. Notably, demographic studies of severe clinical manifestations have revealed a significant skew toward people with pre-existing medical conditions, elderly/senior adults (older than 65 years of age), and immunocompromised individuals, as described below [15,16]. 

### 2.1. Age Vulnerability

The epidemiological studies found age-related death risk to be the most significant factor in SARS-CoV-2 infection, reflecting underlying health conditions and comorbidities. The pathophysiology is attributed to a weakened respiratory immune system, the age-related depletion of the thymic population, and the inability to mount adaptive T-cell immune responses in a timely manner [17]. In unvaccinated individuals, innate immune cells are usually the first line of defense against SARS-CoV-2. In the absence of proper regulatory feedback from adaptive immune cells (T-cells in particular), the responses are rerouted onto the uncontrolled proinflammatory pathways leading to the systemic circulation of inflammatory mediators and the cytokine storm described below. Additionally, inherited immune gene variants may contribute to poor T-cell memory of viral antigens resulting in reinfections and adverse reactions to vaccination. Together, these factors predispose elderly patients to ARDS, cytokine storm, or cytokine release syndrome [18,19]. 

Prior to the implementation of COVID-19 vaccines, the protective efficiency of other booster vaccines, such as bacille Calmette–Guérin (BCG) or flu vaccines, were tested in elderly population clinical trials (e.g., NCT04475302). Interestingly, booster BCG or flu vaccines seemed to help to reduce morbidity and mortality in elderly individuals [20,21]. As soon as the first COVID-19 vaccines became available, the elderly population was prioritized for vaccine administration. 

### 2.2. Immunocompromised Population

Although children younger than 12 generally do not develop severe disease, immunocompromised individuals and some newborns are particularly susceptible to SARS-CoV-2 infection. These individuals carry rare genetic variants that impact the immune system and render them susceptible to SARS-CoV-2 infection. There are more than 200 known genetic defects that can cause an inborn error of immunity, such as primary immunodeficiency (PID) conditions that include rare, severe combined immunodeficiency (SCID) groups [22,23]. The overwhelming majority of PIDs are autosomal-recessive defects of innate or adaptive immune branches, which are subdivided on a gene-type basis (e.g., inborn errors of type I/II interferons and interferon-response/regulatory (IFIH/IRF) pathways) [24,25,26]. Other hereditary conditions, such as cystic fibrosis and hemoglobin S-, C-, or E-beta thalassemia, make individuals susceptible to upper/lower respiratory tract infections, including COVID-19 [27,28]. Often, these individuals cannot be vaccinated for so-called medical reasons. Thus, non-medical interventions, such as social distancing and social isolation, were vital measures for patients with immune deficiency disorders during the COVID-19 pandemic. Although the PID group was exempted from vaccination, the development of a new generation of mRNA vaccines allowed the NIH to initiate clinical trial testing of the Moderna Inc. mRNA vaccine in individuals with PID antibody deficiency disorders [29]. The results have yet to be reported. Omics studies of individuals who have preexisting genetic alterations in the molecular pathways, described in this review, have shed more light on our understanding of genomic susceptibility to SARS-CoV-2 and the immunopathology of the disease. 

People with secondary immunodeficiency states, such as those with late-stage HIV infections, patients who are immunocompromised due to chemo/radiotherapy (e.g., cancer patients), or transplant patients, are also referred to as vulnerable populations that are at risk of dying from SARS-CoV-2 infection [30,31]. The burden of opportunistic infections in this population was also determined to be a risk factor for poor COVID-19 outcomes [32]. Vaccine administration in these groups of populations has been a controversial subject, but preliminary data show that the benefits of vaccination outweigh the risks [33,34]. Individualized, wearable devices with symptom-tracking apps became particularly useful for monitoring early symptoms of infection in the cohort of vulnerable demographic populations [35]. Digital tools, such as smartwatches and smartphone apps, became an essential part of preventive medicine, not only by rapidly identifying viral illness hotspots but also by helping to identify the early symptomatology of COVID-19 based on vital signs and self-reported symptoms [35]. 

### 2.3. Pregnant Women and Individuals with Chronic Conditions

There have been several concerns among pregnant women who contracted the SARS-CoV-2 infection and gave birth. The first concern was whether the infection affected fetal development transplacentally; the second was whether vertical transmission results in newborn infection during delivery, and the third was whether pregnant women are more susceptible to COVID-19 and thus experience more severe outcomes [36,37]. None of these concerns have been corroborated in research studies. Although we have limited data, results have shown that COVID-19 outcomes in women during pregnancy are no more severe than in the non-pregnant population [38]. The majority of studies have reported that babies are born healthy, and immunosuppression during pregnancy helps to avoid a cytokine storm during COVID-19 [39,40]. Furthermore, three FDA-authorized COVID-19 vaccines (J&J, PBNI, and Moderna) demonstrated safety and were approved for use in pregnant and lactating women [41]. Detailed clinical considerations can be found on the CDC website [42].

Another group of vulnerable populations are those with chronic inflammatory/autoimmune conditions, metabolic disorders (e.g., obesity or type II diabetes), and cardiovascular diseases. These individuals are at risk for severe COVID-19 due to hyper-cytokinemia and coagulopathies, the molecular bases of which are described below. The CDC COVID-19 Response Team published several reports on the prevalence of life-threatening multisystem inflammatory syndrome (MIS) in children and adolescents with chronic conditions [43]. Fifty rare genetic variants were identified in 38 genes in association with MIS in young patient cohorts through genomics studies [44]. 

## 3. Genomics and Epigenomics of Host Susceptibility to COVID-19

Sequence analysis of the whole viral genome prompted the discovery of virulence factors, mechanisms of replication, and immunogenicity, and most importantly, it helped to identify host susceptibility factors using high throughput omics approaches. 

### 3.1. Host Genomics

The responses mounted by immune cells involve changes in the expression of a large number of genes, each of which has numerous single-nucleotide polymorphisms (SNPs). The clinicaltrials.gov website has registered 177 active clinical trials that use whole exome sequencing or whole genome sequencing to identify genetic biomarkers of predisposition to severe forms of COVID-19 and the genetic basis of COVID-19 severity (ClinicalTrials.gov) [45,46]. The goal of these studies is to identify very rare germline variants, genetic susceptibility factors, or genetic protective factors in relation to disease severity [47]. In the study published by the COVID-19 Host Genetics Initiative, multiple genes were identified that confer either risk of hospitalization or a protective genotype against hospitalization [48]. The identified gene variants can be linked to other omics data and combined into targeted pathways (e.g., viral particle clearance pathway genes). Utilizing predictive models, a complex risk score can be generated to develop a prognosis for COVID-19 outcomes [49]. 

It is hypothesized that susceptibility to severe COVID-19 is genetically driven. Genome-wide association studies (GWAS) have identified polymorphisms in two main gene clusters that were found to be associated with severe COVID-19: one comprising a cluster of genes involved in the regulation of the immune response; and the other determining the ABO blood groups [50]. Certain HLA haplotypes (e.g., HLA-DRB1*04:01) were found to be associated with severe SARS-CoV-2 infection, while other haplotypes containing HLA-C alleles from group C1 were frequently associated with mild COVID-19 cases [51,52,53]. A recent meta-analysis of several GWAS studies identified significant associations of APOE, ACE1, TMPRSS2, CCR5, and HLA immune gene polymorphisms with a predisposition to severe COVID-19 [54,55]. Two loci on chromosome 11q23.3 and 11q14.2 were identified to be enriched in critically ill COVID-19 patients, suggesting that these locations are sites of gene susceptibility to severe forms of the disease [56]. However, it is known that GWAS largely depend upon the studies’ sample sizes. For example, the first COVID-19 GWAS finding for ABO blood groups (from an Italian outbreak of 600 cases) identified patients with blood type A as being at higher risk for severe COVID-19 and individuals with blood type O as having a very low risk of severe disease. Subsequent studies that included 8000 participants concluded that ABO groups were not associated with COVID-19 incidence or severity. Rather, it is locus 3p21.31, containing more than 100 cytokine/chemokine genes and the novel therapeutic target gene LZTFL1, that has several single-nucleotide polymorphic risk factors [57,58]. Genotyping of a small group of patients with COVID-19-associated coagulopathies identified a set of risk SNPs in the vWF and ADAMTS13 genes [59]. Additionally, two APOL1 haplotypes (G1 and G2) were found to be associated with rapid progression to COVID-19-associated nephropathy and end-stage renal disease [60]. For a specific focus on the topic of allele susceptibility and genetic polymorphic risk factors for COVID-19 severity, we refer readers to a series of comprehensive reviews with detailed tables and figures [61,62,63,64]. 

Mutations in the interferon pathways were perhaps the most valuable diagnostic discoveries in patients with rare inborn errors of IFNI-III, IFN-response genes (e.g., IFIHs, IRFs), and STAT kinases. Almost all of them were described in case reports. Many research reviews produced lists of rare monogenic variants identified in patients with severe/critical COVID-19, including multisystem inflammatory syndrome in children (MIS-C), in which the above-mentioned interferon pathways took a leading place [44,65]. Although much work remains to be done to establish the usefulness of GWAS for clinical care and actionable public health measures, the precision medicine field is moving toward genotyping individuals at birth to identify genetic risk factors for existing and newly emerging infections [66,67]. 

### 3.2. Host Epigenomics

Epigenetic alterations during virus–host interactions can significantly affect the adequacy and magnitude of both immune and inflammatory responses, influencing clinical outcomes of SARS-CoV-2 infection [68,69]. Whole genome bisulfite sequencing (WGBS) is a next-generation sequencing technology that allows users to analyze host DNA methylation at a single base resolution and determine the prognosis of the disease. There is an active search for epigenetic and proteomic biomarkers that may help to stratify the risk of severe respiratory outcomes in COVID-19 patients [69,70]. For example, differential expression of methylation-sensitive genes, such as type I interferon genes, angiotensin-converting enzyme 2 (ACE2), and IL6, predisposes patients’ antiviral responses to be exaggerated [71,72]. A higher rate of HLA-C methylation in severe COVID-19 patients was identified compared to moderately ill patients, corroborating the GWAS described above [73]. Additionally, epigenetic alterations in hypoxia-inducible factor 1 (HIF-1) signaling, Toll-like receptor signaling (TLR), fatty acid oxidation/degradation, autophagy, retinoic acid-inducible gene I (RIG-I) signaling, and demethylation of IL17 signaling genes may predispose certain patients to fatal outcomes [74]. These changes can be reversed by pharmacological agents known as ‘epidrugs’, thus protecting certain individuals from the development of a cytokine storm [70,75]. MicroRNAs represent another therapeutic and diagnostic opportunity to modulate anti-viral host immune responses to SARS-CoV-2, some of which are under research and investigation in clinical trials (NCT04583566 [76]). Epidrugs have not been widely tested in infectious disease settings, but the idea of temporarily resetting target gene expression to prevent activation of defective immune pathways is under extensive investigation [77]. 

## 4. Individual Changes in Metabolome, Lipidome, and Microbiome during SARS-CoV-2 Infection

### 4.1. Host Metabolomic Response

Serious metabolic disorders are generally very rare and hereditary in nature. Commonly acquired metabolic diseases, such as type II diabetes, obesity, or hypercholesterolemia, are pre-existing conditions that put individuals at risk for severe COVID-19 outcomes. 

Several research studies aimed to classify COVID-19 severity according to metabolomic profiles (i.e., mild versus severe) and predict potential outcomes. Earlier studies identified 20 abnormal metabolites within pyrimidine, tryptophan, aspartate, phenylalanine, and asparagine pathways that correlated with the severity of ARDS in patients’ serum [78]. Increases in metabolic markers of oxidative stress (methionine sulfoxide, cystine), proteolysis, and renal clearance dysfunction (creatine, creatinine, polyamines) were correlated with concentrations of IL6 and renal failure (another severe outcome of COVID-19) [78,79]. 

Large-scale plasma analysis revealed molecules associated with the host response to SARS-CoV-2. Alterations in 77 plasma metabolites including, lipids, polyamines, and sugars, as well as their derivatives, were detected in critically ill COVID-19 patients compared to those with mild cases of the disease [80]. Increased levels of phenylalanine, glycoproteins, mannose, 3-hydroxybutyrate, and isoleucine and decreased levels of citrates have been observed in severe COVID-19 subjects compared to mild or asymptomatic cases [81]. There has been a trend toward increased triglyceride concentrations and a decrease in low-density lipoprotein fractions in the plasma of patients with moderate-to-severe COVID-19 disease compared to post-COVID patients [81,82]. 

Several classification models have been applied to specific metabolite changes in conjunction with clinical data to decipher biochemical markers of COVID-19 severity. Lipidomic profiles help to sequestrate critical COVID-19 illness from mild disease based on high serum levels of LDL cholesterol, total cholesterol, arachidonic/oleic acids, ceramides, and phosphatidylcholines/phosphatidylethanolamines [83,84]. To predict patients’ infection severity, untargeted metabolomics and Monte Carlo algorithms were used in [78], identifying deoxycytidine, ureidopropionate, kynurenine, and multiple short-chain acylcarnitines as predictors of COVID-19 severity.

Although metabolic profiling has not entered clinical practice, in the future, it may provide a means to better diagnose and classify COVID-19 susceptibility and predict severity. For example, individuals who were diagnosed with preexisting metabolome conditions, such as chronic vitamin D deficiency, particularly adults 65 years and older, were recommended to increase their vitamin D consumption to prevent severe forms of COVID-19 (e.g., those accompanied by increased intravascular coagulation) [85,86]. Dietary supplementations with other vitamins, nutraceuticals, and micronutrient minerals were also recommended for the general population, particularly for individuals with known deficiencies [87]. Identification of defective metabolic pathways responsible for increased susceptibility to severe outcomes and complications is now part of the precision diagnostics portfolio (Figure 1). In the near future, it will facilitate the development supplementary management protocols and the navigation of the repositioning of existing drugs to improve COVID-19 outcomes.

### 4.2. Microbiomics 

There are intricate interactions between human microbiota and infectious diseases. SARS-CoV-2 affects the human microbiome with the infection causing diarrhea in some patients [88,89]. Although not directly studied, the microbiome of the lungs also seems to be severely disturbed in ARDS patients, spinning into the pro-inflammatory state and making the lung ARDS’s target organ [90]. Probiotic prophylaxis (although not an individualized approach to treat/prevent ARDS) was recommended during the pre-mass-vaccination period at the beginning of the pandemic with the idea that “something is better than nothing” [91,92,93,94]. 

Interestingly, an immunization response signature has been recently found in commensal gut microbiota: the gut microbial fucose/rhamnose degradation pathway was found to be positively correlated with activation of AP-1 and enzymes producing PGE2 mRNA expression in the blood of newly vaccinated individuals [95]. The link between this microbiome composition and the host’s genetics and epigenetics is just beginning to emerge in the cross-ome correlations of molecules and patient prognoses. Perhaps in the future, microbiomics will provide diagnostic and prognostic value for the assessment of precision drug effectiveness. 

## 5. Transcriptomics and Proteomics of SARS-CoV-2 Infection 

### 5.1. Host Transcriptomics

Host transcriptome analysis of immune cells and diseased tissues has been an integral part of precision diagnostics. The discovery of transcriptomic immune response biomarkers early in the disease course has been particularly important for vulnerable populations. SARS-CoV-2 infection occasionally results in a critical illness that begins with uncontrolled overexpression of proinflammatory cytokines (the so-called cytokine release syndrome or cytokine storm) [96]. Clinically, these molecular events manifest as acute respiratory distress syndrome (ARDS), which may lead to multiple organ failure and death [97]. 

The SARS-CoV-2 virus is recognized by the innate immune system and elicits a distinct transcriptional gene expression pattern driven by mono- and poly-morphonuclear cells [98,99,100]. These receptors (e.g., PRRs, TLRs, or cGAS-STING) trigger the upregulation of downstream pathways, activating the cellular inflammatory signalosome [101,102,103,104]. Abnormal upregulation of inflammasome components, HMGB1, and oxidative stress response transcripts and proteins has been recently demonstrated in biomarkers of fatal ARDS in COVID-19 patients [105,106]. Additionally, strong activation of IL6 and IL17 signaling pathways, NF-kB, AP-1, JAK1,2,3, MAPKs, p38, and ERKs have been reported to underlie pathological dysregulation of the immune system during severe COVID-19 disease [107,108,109,110]. These precision diagnostic biomarkers have become the omics targets for drug candidates tested in clinical trials, some of which are described in Section 7. 

Several predictive biomarkers of ARDS have already been identified in peripheral blood [15]. For example, low expression of interferon-gamma and interferon-response genes pathways were identified as baseline risk factors for subsequent cytokine release syndrome during COVID-19 [15,111,112]. The significantly reduced expression of the inflammation suppressor IFN I- and IFN III-type genes and the overexpression of monocyte- and granulocyte-derived hyper-inflammatory cytokine transcripts (e.g., GMCSF) at the first symptoms of the disease were proposed to predict COVID-19 mortality [113,114,115]. Transcripts that encode the main effectors of the IFN I type-mediated antiviral response and interferon-stimulated genes (ISGs) (e.g., IFI44L, IFI27, RSAD2, SIGLEC1, IFIT1, ISG15) were downregulated in patients who became critically ill compared to the patients with mild and moderate diseases [116]. Identification of dysregulated interferon pathways prompted the initiation of several clinical trials, implementing adjunctive interferon treatment and GMCSF blockers in patients at risk, as described below. 

Novel transcripts were discovered among molecules that are positively (PKMYT1, HJURP, FOXM1, and HIST1H2BO) or negatively (FGF9, DYRK2, APBB1, NELL2, and P2RY10) correlated with disease outcomes [117]. Additionally, novel biomarkers were detected in neutrophil activation pathways that were highly upregulated in the severe COVID-19 group compared to healthy controls, including CD177, CEACAMs, MMP8, ELANE, OLFM4, Gal-9, DEFAs, and MPO [117,118]. Single-cell transcriptomes revealed the clonally expanded proliferation of myeloid immune cells with the transcriptomic signature of so-called exhausted T-cells [118,119,120]. Additionally, single-cell B-lymphocyte transcriptomics revealed the expansion of B-cells with characteristic gene expression of plasmablasts, which was confirmed by proteomic assessment of surface receptor molecules. However, plasmablasts of patients with severe and critical illness failed to produce IgA2 compared to asymptomatic patients [120]. 

Numerous Gene Ontology term (GO) signatures were consistently found in the peripheral blood transcriptome of severe COVID-19 cases by several investigators. These signatures included neutrophil degranulation, platelet activation/degranulation, blood coagulation defects/blood vessel damage, and acute phase response [121,122,123]. Similar transcriptome signatures were found to be activated in various fatal conditions, such as immune reconstitution inflammatory syndrome (IRIS) and sepsis [124,125,126,127]. 

High throughput transcriptomic methodology is a precision diagnostic procedure that has the fastest turnaround time among all omics. The caveat to utilizing transcriptomic biomarkers from peripheral blood is that they reflect systemic inflammation and are very dynamic in nature [128]. Thus, the precise time of sample collection can be a challenging task. Cytokine mRNAs are expressed transiently in the immune cells that infiltrate the focal sites of inflammation, and the so-called spillover into systemic circulation occurs in a very severe disease state [129,130]. Hence, to achieve a precision diagnostic level, the transcriptomic biomarkers need to be identified in other biological fluids, for example, in bronchoalveolar lavage or nasopharyngeal swabs in patients with suspected pneumonia [109]. 

### 5.2. Host Proteomic Response 

Assessment of the proteome in patients with COVID-19 of various severity levels identified several distinct proteome signatures that predict the individual response to infection and disease outcomes. This finding was incredibly important for identifying a window of opportunity for therapeutic interventions. 

Proteins associated with the excessive inflammatory response (C-reactive protein, CRP), increased blood coagulation (D-dimer), and cell damage (lactate dehydrogenase) have been identified as predictors of COVID-19 severity or mortality [131]. Targeted plasma proteomics identified unique inflammatory mediator profiles in hospitalized COVID-19 patients (those treated in intensive care units or not) [132]. Among them were circulated cytokines/chemokines (TNFα/TNF receptors, GMCSF, CXCL10, CXCL11, and CCL19), complement-cascade factors (C2, SERPINA5, CR2, C1QTNF1, F2), and matrix metalloproteinases (MMP1, MMP7) and their inhibitor 1 (TIMP1), as well as galectin-9, CD46, CD40, VEGFA, and thrombomodulin [132]. Moreover, receiver-operating characteristics (ROC) curve analysis showed diagnostic effectiveness for separating critically ill patients from moderately ill patients and from the rest of the patients during the course of the disease and up to 75 days after symptom clearance [132]. Eleven plasma proteomic biomarkers of ARDS were identified in several studies, including cytokines (e.g., IL6), components of complement, neutrophil degranulation, and platelet aggregation [133,134,135]. Most of these biomarkers were also identified in the transcriptomic studies described above, suggesting high concordance between two precision diagnostics. 

High throughput fluorocytometric proteomics methods have provided very informative immune cell surface protein phenotypes and quantitative assessment of antibody responses to SARS-CoV-2 in plasma [136]. Alterations in numerous important pathways were found in individuals with severe COVID-19, which were broadly described as an immune response (cytokine/receptor interactions and complement), the coagulation and platelet degranulation pathways, and lipid/amino acid metabolism enzymes [137]. However, when individual biomarkers were assessed, significant heterogeneity was noted among studied patients. 

A recent proteomic study identified 42 distinct plasma biomarkers attributed to the acute and convalescence phases of COVID-19 compared to sepsis as a complication of community-acquired pneumonia [138]. Proteomic biomarkers of long COVID were also identified with a natural language processing algorithm (NLP) and UniProt Knowledgebase mining. Major organ/body systems and cell type disfunctions were attributed to lymphatic, digestive, and nervous system proteomes [139]. Based on these signatures, a list for drug repositioning was proposed to mitigate long-COVID symptoms.

Numerous confirmatory studies have followed high throughput screening proteomics, including studies of damage-associated molecular pattern (DAMP) molecules, such as galectin-9 (Gal-9). The proteolytically cleaved form of the Gal-9 protein showed potential to become an additional (to D-dimer and CRP) diagnostic marker but specific to assessing COVID-19 severity [140,141]. Moreover, it was observed in the plasma of patients with COVID-19 pneumonia that the N-cleaved form of Gal-9 decreased significantly in response to the anti-IL6 drug tocilizumab [141]. 

Future directions in this field should be focused on improving proteomic-guided differential diagnosis, quantitatively assessing disease severity, and predicting the course of disease progression to assist in personalized therapy decision-making. To achieve these goals, the International Severe Acute Respiratory and Emerging Infections Consortium (ISARIC) reported COVID severity scores, based on plasma proteomics, clinical lab tests, and drugs prescribed for patients with COVID and other comorbidities [142]. The candidate drugs to treat severe complications or to prevent the progression of the disease were proposed based on drug–protein interaction analyses, some of which are described in Section 7 of this report. 

## 6. Multiomics and Cross-Ome Comparisons

### 6.1. One Patient Multi-Omics

As described above, the dysfunctional blood immune response signatures were identified as diagnostic by many interindividual transcriptomics, proteomics, or metabolomics studies [109]. Comprehensive one-patient multiomics studies have also been performed. One such study identified more than 17,000 biomolecules, which included transcripts, proteins, metabolites, and lipids that are correlated with severity of COVID-19 [121]. Multiomics profiles reveal severe neutrophil dysfunction (aka neutrophil extracellular traps), which led to repurposing of the drug colchicine and corticosteroid inhalation regimens to treat patients at risk for severe COVID-19 illness, resulting from hyperinflammation [143,144]. Another in-depth multi-omics profile identified nearly 26,000 biomolecules and highlighted the main signatures of T-cell exhaustion, neutrophile dysfunction, and accelerated tryptophan catabolism in critically ill COVID-19 patients [145]. Therapeutic interventions with indoximod and navoximod were proposed to mitigate the affected tryptophan–kynurenine metabolic pathway [145]. 

While identifying non-invasive markers of target organ inflammation (in the lungs), re-searchers discovered that salivary IL19 mRNA expression was significantly correlated with COVID-19 severity [146]. In the same multiomics study, plasma levels of IL19 were measured in response to immuno-modulating treatments with corticosteroids, tocilizumab, and IFNβ, revealing that IFNβ treatment was more effective at reducing IL19 and overall signs of inflammation [146].

Another study produced a large-scale, longitudinal multi-omics dataset from severe COVID-19 patients. High frequencies of distinct HLA types, longitudinal changes in T/B-immune-cell transcriptomics, and changes in 200 plasma protein concentrations have been reported from samples collected during the course of the disease [147]. Urine metabolic profile results have yet to be made public (for more information, see https://nih.go.kr/biobank, accessed on 6 June 2023).

Schultheiß et al. analyzed several single-cell multiomics studies from the blood and tissues of 318 patients suffering from symptoms of long COVID-19 post-acute sequelae [148]. The researchers concluded that IL1β, IL6, and TNFα axes are involved in long COVID-19 symptomatology and can be treated with precision drugs, as outlined Section 7.

Comprehensive, one-patient multiomics studies have been scarce due to the cost and complexity.

### 6.2. Cross-Ome Bioinformatic, Data Mining and Analytics

Precision diagnostics relies on big analytics platforms to deliver actionable data in a format that supports clinical decisions. The big data analysis platforms based on artificial intelligence algorithms were designed to interpret multi-omics data within accelerated timelines [2]. Combining individual omics data with demographical/ethnic data and prior health conditions (patients’ phenomes) allowed for the identification of genes and molecular pathways involved in COVID-19 hospitalization cases [142,149]. Strong associations were identified between multiome biomarkers and individuals’ pre-existing conditions, such as kidney disease, ischemic heart disease, cerebrovascular disease, and hypertension [150,151].

Large databases containing metadata and omics datasets, generated from the same type of samples, allowed for data mining and visualization in the Shiny app (https://db.combat.ox.ac.uk, accessed on 6 June 2023) [152]. Studies that analyzed individual omics data found significant alignment between various omes, combining them into COVID-19 signatures and classifications (Figure 1). Numerous bioinformatics project results have been reported from multi-omics studies, linking biomolecules or changes in their abundances to COVID-19 outcomes. Several of them have been published on GitHub (https://github.com/ijmiller2/COVID-19_Multi-Omics, accessed on 6 June 2023) and in the peer-reviewed scientific literature [153,154].

Machine learning multiomics models (e.g., calculation of the area under the receiver operating characteristic curve, AUC) predicted patients’ survival with 92% accuracy, based on 10 proteins and five metabolites [155]. Combining single omics datasets from various studies, machine learning models achieved high accuracy scores for negative and positive classifications of COVID-19 diagnosis, as well as COVID-19 severity prediction, imputing complex molecular interaction among diverse molecules [156]. Moreover, the machine learning approach was successful in constructing and validating nomogram models for multiomics parameters, which predicted patients’ admission to intensive care units with 90% accuracy [157].

Computational analyses of transcriptomic data from blood and lung biopsies concluded that drug target searches should be performed separately in the pool of upregulated and downregulated mRNAs [158]. For example, immunosuppressive drugs can be utilized for overexpressed cytokine genes, and under-expressed molecules (MRPL41, COL1A1, PLEKHF1, SPOCK3, FSCN1) can be targeted with drugs for specific pathways associated with the above-mentioned molecules (e.g., dosulepin, mexiletine, bromopride, fenoprofen, tetrahydroalstonine, and cytisine) [158].

Machine learning classification models, multivariate models, pathway enrichment analyses, tree-based ensemble machine learning methods, and dimensionality reduction multi-omics-based models were found to be the most popular in the identification of unique patterns and signatures of COVID-19 disease in the general population and in the vulnerable population subgroups described above. Together, multiome-based applications have great potential in precision diagnostic medicine (Figure 1).

## 7. Precision Medicine: Repurposed and Novel Drugs for COVID-19 Patients

Omics data have become an essential part of diagnostic medicine and have served as the basis for discoveries in precision and preventive medicine. Medical institutions initiated clinical trials of repurposed drugs, prompting pharmaceutical companies to begin development of novel therapeutics and vaccines [159,160,161].

Precision therapies can be divided into two large groups: those directly against the SARS-CoV-2 virus and those that modulate host immune responses. The control of disease progression is dependent upon both drug groups; thus, multiple combinations of drugs have been tested, as summarized below.

### 7.1. Precision Antiviral Drugs and Monoclonal Antibodies

Individualized antiviral drugs aim to decrease viral load and accelerate viral RNA clearance, thus indirectly acting as immunomodulators and preventing the damage caused by exaggerated immune responses to unknown viral pathogens.

The first FDA-approved antiviral treatment, remdesivir (Gilead^®^), is being safely used in elderly adults, pediatric populations, and patients with hereditary hyper-coagulopathies or thrombophilia [162,163,164]. The oral treatment molnupiravir (Merck^®^) was found to be effective in the pre-hospitalization stage of COVID-19 [165]. Emergency-authorized ritonavir-boosted nirmatrelvir is prescribed to older adults (age > 65 years old) or patients with an underlying health condition, such as heart diseases, cancer, diabetes, or obesity [166]. As these patients take various types of medications daily, physicians and pharmacists need to carefully consider possible drug–drug interactions. Another oral drug, Paxlovid (Pfizer^®^), consists of a combination of nirmatrelvir and ritonavir. Paxlovid has been FDA approved in pregnant and lactating women who have contracted SARS-CoV-2 infection [167]. Additionally, treatment with Paxlovid reduces the occurrence of long COVID-19 and post-acute sequelae of severe SARS-CoV-2 infections [168]. Generally, it is advised to begin taking antiviral drugs early in the disease course, as clinical trials have shown that they had no significant effect on patients with severe COVID-19 who were already being ventilated [169]. Thus, it is extremely important to identify and target vulnerable populations who are at risk for severe outcomes to initiate early treatment.

Although not described in this review, the structure and conformation of neutralizing monoclonal antibodies (MABs) are routinely tested in genomic, transcriptomic, and proteomic studies [170]. The MABs are FDA-authorized treatments for at-risk patients with clinical progression to severe COVID-19 disease, and they are designed against the viral spike protein [171]. Several antibody drugs (e.g., bamlanivimab and etesevimab) have been tested in immunocompromised and other at-risk-group patients, with positive outcomes, such as reduced viral load and decreased illness duration (or mortality) in inpatient and outpatient settings [171,172]. However, newly emerging lineages of SARS-CoV-2 like XBB/XBB.1.5 have already shown so-called immune resistance to existing monoclonal antibody treatments, as well as immune escape from humoral immunity elicited by four-dose vaccination regimens [173].

### 7.2. Immunomodulators Targeting Host Responce

Prolonged hyperinflammation has very dangerous consequences; hence, a number of immunomodulators and anti-inflammatory drugs have been proposed for use in hospitalized COVID-19 patients based on multi-omics research results. Pegylated interferon lambda is being tested as a precision immunomodulatory drug in clinical trials to improve the immune response to SARS-CoV-2 [174]. Other interferons have also been tested (with or without antiviral, glucocorticoid, and nonsteroid anti-inflammatory drugs) and have been shown to reduce the risk of mortality (Table 1) [142,175]. The cytokine–cytokine receptor interaction (e.g., TNFα/TNFR), JAK-STAT, and other stress response kinases have been targeted with precision drugs, as they were most significantly associated with poor COVID-19 outcomes in transcriptomic and proteomic studies [176].

Systemic upregulation of the cytokines IL6, GMCSF, TNFα, and NLRP3, identified in transcriptomic and proteomic studies, is noteworthy to mention here, as these cytokines are targetable molecules for drugs such as adalimumab, tocilizumab, anakinra, and RAPA-501-Allo (Table 1) [177,178]. Various combinations of targeted therapies with precision antiviral drugs are the subjects of both ongoing and completed clinical investigations, e.g., comparison of tocilizumab and baricitinib and combinations of interferon-β1a/β1b or tocilizumab—with lopinavir/ritonavir [179,180,181]. The combination of corticosteroids and precision drugs (such as anti-IL6/IL6R antibodies) has been reported to decrease ventilator-free time and 28-day mortality in critically ill patients who were admitted to the intensive care unit [182].

**Table 1:** Clinical utility of precision drugs against biological targets identified in omics studies.

Omics Target	Drug(s)	NCT	Efficacy/Outcomes	Reference
IL6R	Tocilizumab	NCT04381936	Reduction in mortality in patients with high-baseline IL6	[183]
	Tocilizumab	IRCT20150303021315N17	Reduction in the risk of death when treatment begins in early stages of respiratory failure	[184]
	Tocilizumab	Retrospective analysis	Shortened time to hospital discharge from intensive care unit regardless of the respiratory support	[185]
	Sarilumab	NCT04327388	Did not improve survival and ventilatory outcomes in critical patients (study was underpowered)	[186]
IL6	Siltuximab i.v.	NCT04322188	Downregulated IL8 and pentraxin 3 in the blood; reduced mortality in patients on ventilation support	[187]
IFNB1a	Nebulized interferon-β1a alone	NCT04732949	Reduced mortality but not in-hospital time or time to full recovery	[188]
	Nebulized interferon-β1a with antivirals	NCT04343768	Shortened time to significant clinical improvement	[181]
INFAb	Nebulized interferon-α1a	Retrospective analysis	Associated with reduced mortality and accelerated recovery	[189]
IFNB1a versus IFNB1b	Interferon-β1a s.c. or Interferon-β1b	NCT04343768	Significant reduction in time to clinical improvement (TTCI) in IFNβ1a arm	[181]
NLRP3	Tranilast	IRCT20200419047128N1	Reduced length of hospitalization and mortality	[190]
IL1β	Canakinumab	Case-control study	Decreased need for mechanical ventilation; earlier hospital discharge	[191]
IL1R	Anakinra i.v.	NCT04318366	Associated with clinical improvement in 72% of patients	[192]
		NCT04357366	Lowered incidence of severe respiratory failure; increased survival rate	[193]
JAK 1/2	Baricitinib	NCT04381936	Reduced mortality in hospitalized COVID-19 patients	[194]
	Baricitinib with antivirals	NCT04401579	Improved clinical status among patients on high-oxygen or noninvasive ventilation support	[195,196]
JAK 1/3	Tofacitinib	NCT04469114	Lowered risk of death and/or respiratory failure	[197]
TNFα	Adalimumab	GRA physician registry	Decreased odds of hospitalization in patients with rheumatic disease treated with adalimumab	[198,199]
	Infliximab	ISRCTN40580903	Inferior to namilumab	[200]
GMCSF	Namilumab	ISRCTN40580903	Significant reduction in inflammation in hospitalized COVID-19 patients	[200]
	Lenzilumab	NCT04351152	Improved survival without invasive mechanical ventilation in hospitalized patients with COVID-19	[201]
TKI	Nintedanib	jRCTs051200036	Shorter length of mechanical ventilation Reduction in pulmonary fibrosis on imaging tests	[202]
CD6	Itolizumab	CTRI/2020/05/024959	Improved lung oxygenation parameters and blood cytokine profiles in ARDS patients	[203]
IL17	Netakimab	NCT05302947	Lowered mortality rate; shortened time to hospital discharge; superior to baricitinib	[204]

NCT, National Clinical Trial number. Drug classes: -mab-, neutralizing monoclonal antibodies (biological drug), usually administered s.c., subcutaneous injections. -ib-, -ept-, inhibitor drugs. -ast-, antagonist drug. -nra-, receptor blocker, usually administered s.c. or i.v., intravenous injection.

Several other clinical trials, not outlined in Table 1, did not find statistically significant differences in short-term survival between study groups for two main reasons: either the studies were too small (underpowered) or they accepted very sick patients on the basis of compassionate care, when immunomodulators were unable to reverse the devastating consequences of ARDS or MIS-C [205].

In contrast, it has been identified that, in patients with severe pneumonia, IL6 blockade may be effective in the late disease stage. Thus, the timing of precision drug administration should be evaluated and incorporated into personalized healthcare decision-making modules [206].

There is ongoing research in the cohort of patients who have already been treated with precision drugs for various chronic diseases and who contracted COVID-19 (or received vaccination). This topic is beyond the scope of our review, but one example is presented in Table 1 [199]. The use of the immunomodulator adalimumab by patients with rheumatoid arthritis decreased the odds of hospitalization with severe forms of COVID-19 [198].

In summary, before prescribing any precision medicine, it is important to know the gene expression profiles and proteome/metabolome composition unique to the individual patient.

## 8. Preventive Medicine: Vaccination 

Precision vaccination against SARS-CoV-2 has proved to be the most successful public health effort to stop the COVID-19 pandemic [170]. The successful vaccination campaigns, particularly with the mRNA types from Pfizer and Moderna, have significantly reduced the numbers of hospitalizations and deaths worldwide [207,208,209]. The SARS-CoV-2 virus exhibits a relatively fast mutation rate, and reinfection in healthy individuals is common [17,210,211,212]. In the majority of patients, new lineages of SARS-CoV-2 are the cause of reinfection [17,213]. In rare cases, reinfection occurred with the same variant from the same clade (as confirmed by next-generation sequencing) [210,214,215]. As described above, vaccinations are now approved by the FDA for pregnant women at all stages of pregnancy, and they do not pose a risk of fetal miscarriage [41].

### 8.1. Vaccines Breakthroughs

Since mass vaccination, asymptomatic individuals have become major contributors to the ongoing COVID-19 pandemic, as they do not manifest symptoms and are unaware of their infectiousness [16,216]. During the emergence of the Delta variant, the term “super-spreaders” began being used to describe asymptomatic people who exhibit high viral shedding, exposing other individuals to the virus for a longer period of time [217]. Immune gene variants that contribute to poor T-cell memory and underlying vaccine breakthroughs are most likely attributed to the accumulation of mutations in circulating SARS-CoV-2 lineages, such as sub-lineage Omicron (BA.2-BA.5), increasing the number of super-spreaders and the transmissibility of new SARS-CoV-2 variances of concern [1,218,219].

All approved vaccine types encode the truncated SARS-CoV-2 spike protein, generate neutralizing antibodies, and induce virus-specific adaptive T-cell responses against two major clades of SARS-CoV-2 (clade A and B) [220]. Emergence of novel mutations of SARS-CoV-2 led to accumulating evidence of the so-called vaccine breakthrough phenomenon, i.e., increased cases of COVID-19 in patients who were fully vaccinated [221,222,223]. Although in the majority of these vaccine breakthrough cases, the infections were mild to asymptomatic, some people presented with severe disease, perhaps due to the inability to develop sufficient T-cell-mediated immunity after vaccination, caused by genetic variation in immune genes [16].

Advances in viral genomic surveillance and vaccine manufacturing have significantly reduced post-vaccination breakthroughs and so-called post-acute COVID-19 (also long COVID) in local communities and subsequently worldwide [224]. Pfizer’s and BioNTech’s modified bivalent vaccines have been recently approved by the FDA as boosters that target Omicron BA.4/5 lineages [10,225]. It has been noted that double- and triple-booster vaccinations reduce the risk of developing long COVID-19 [8]. Considering the ease of vaccine design alterations, new boosters will be coming on the market in the near future [226,227].

### 8.2. Vaccine Side Effects

New mRNA vaccines are involved in a small but accountable percentage of adverse events, which can potentially be predicted and mitigated with precision drugs [170]. For example, pityriasis rubra pilaris, a rare complication of COVID-19 vaccination, was successfully treated with the interleukin-17A inhibitor ixekizumab [228]. Response to the vaccine is genetically driven. Thus, before administering preventive medicine, it is important to know the patient’s genetic makeup, as it may cause unanticipated vaccine side effects in the form of cytokine release syndrome and cytokine-induced inflammation.

## 9. Summary and Future Directions

Multiomic delineation of host susceptibilities and immune responses to SARS-CoV-2 and COVID-19 severity, as discussed in this review report, reinforced precision diagnostics and precision medicine, in turn empowering healthcare practitioners to choose the most appropriate course of treatment for patients with COVID-19 or to inform vulnerable populations about vaccine safety and other preventive measures.

The future of the rapidly advancing field of omics will be in precision single-cell multiomics and data integration into complex multi-level intracellular signalosome profiles. Implementation of artificial intelligence with advanced neural networks will further shape evidence-based information gathered from multiome and clinical data. This evolution of multiomics will undeniably benefit medicine and public health.

## Data Availability

Data sharing not applicable.

## Abbreviations

ACE2	Angiotensin-converting enzyme 2
ARDS	Acute respiratory distress syndrome
CDC	Centers for Disease Control and Prevention
CEACAM	Carcinoembryonic antigen-related cell adhesion molecule
GWAS	Genome-wide association study
HLA	Human leucocyte antigen
IL	Interleukin
ADAMTS13	A disintegrin and metalloprotease (domain) of metallopeptidase with thrombospondin type 1 motif 13
TLR	Toll-like receptor
Gal-9	Galectin 9
CRP	C-reactive protein
PRRs	Pattern recognition receptors
SNPs	single nucleotide polymorphism
cGAS-STING	cyclic GMP–AMP synthase (cGAS) stimulator of interferon gene (STING)
HIST1H2BO	Histone H2B type 1-O
HMGB1	High mobility group box 1 gene
NLRP3	NLR (nucleotide-binding domain and leucine-rich repeat containing) family pyrin domain containing 3
TMPRSS2	Transmembrane protease serine 2
TCRA/D region	T cell receptor alpha/delta genes
TIMP1	Metalloproteinase inhibitor 1
IFNγ	Interferon gamma
IFIH	interferon induced with helicase C domain gene family
IRF	interferon regulatory factor gene family
PGE2	Prostaglandin E2
MMP	Matrix metalloproteinase
AP-1	Activator protein 1
J&J	Janssen Biotech Inc. and Johnson & Johnson
PBNT	Pfizer-BioNTech
VEGFA	Vascular endothelial growth factor A
GMCSF	Granulocyte–macrophage colony-stimulating factor
JAK 1/2	Janus kinases
SIGLEC	Sialic acid–binding, immunoglobulin (Ig)-like lectin
TKI	Tyrosine kinase inhibitor
IL6Rα	Interleukin receptor alpha
IL1R	Interleukin 1 receptor
AP-1	Activator protein-1
MAPKs	Mitogen-associated protein kinase
ERKs	Extracellular signal-regulated kinase
